# Prognostic value of platelet to lymphocyte ratio in patients with castration-resistant prostate cancer: a systematic review and meta-analysis

**DOI:** 10.3389/fonc.2025.1655520

**Published:** 2025-12-11

**Authors:** Junhui Ying, Changchun Zhou, Yili Jin

**Affiliations:** Department of Urology, Dongyang Hospital Affiliated to Wenzhou Medical University, Jinhua, Zhejiang, China

**Keywords:** castration-resistant prostate cancer, platelet-to-lymphocyte ratio, prognosis, overall survival, progression-free survival

## Abstract

**Introduction:**

Although the platelet-to-lymphocyte ratio (PLR) has been identified as a prognostic marker in various cancers, its role in castration-resistant prostate cancer (CRPC) remains uncertain. This meta-analysis examines the prognostic significance of PLR in relation to overall survival (OS) and progression-free survival (PFS) in patients with CRPC.

**Methods:**

We systematically searched PubMed, Embase, Web of Science, and the Cochrane Library up to March 11, 2025. Two reviewers independently screened studies, extracted data, and assessed quality using the Newcastle–Ottawa Scale (NOS). Pooled hazard ratios (HRs) with 95% confidence intervals (CIs) were calculated using a random-effects model. Sensitivity and subgroup analyses explored heterogeneity and assessed result stability. All analyses were performed using Review Manager 5.4 and STATA 15.0.

**Results:**

A total of 13 studies (14 comparison groups; 2,405 patients) were included. High PLR was significantly associated with shorter OS (HR = 1.62, 95% CI: 1.30-2.03), but not with PFS (HR = 1.25, 95% CI: 0.92-1.69). Subgroup analyses confirmed the association with poor OS in prospective studies, patients aged ≥72, European populations, those on hormone therapy, and studies using a PLR cut-off ≥150. Heterogeneity mainly arose from differences in study design, treatment, and region. Sensitivity analyses and Egger’s test confirmed the robustness of findings with no publication bias.

**Discussion:**

PLR is a significant predictor of OS in CRPC and may help guide clinical risk stratification. However, its role in predicting PFS is limited. Further prospective studies are needed to validate its clinical utility.

## 1 Introduction

Prostate cancer (PC) represents the most frequently diagnosed malignancy and ranks as the second most common cause of cancer-related death among men worldwide (1). Castration-resistant prostate cancer (CRPC), which involves disease progression despite castrate levels of serum testosterone (<50 ng/dL), manifests as rising prostate-specific antigen (PSA), radiographic advancement, or symptom deterioration (2). As the terminal stage of PCa, CRPC is responsible for the majority of PCa-related deaths, with 10%–20% of patients developing CRPC within five years of initiating androgen deprivation therapy (ADT) (3). Although therapeutic strategies such as taxane-based chemotherapy (docetaxel, cabazitaxel), androgen receptor inhibitors (abiraterone, enzalutamide), and radiopharmaceuticals (radium-223) have been introduced, overall survival in CRPC remains limited, averaging 13–16 months (4–6). These findings underscore the need for reliable prognostic biomarkers to enhance risk stratification and inform clinical decision-making.

Recent studies suggest that systemic inflammation plays a crucial role in the progression of CRPC and its resistance to treatment (7). Chronic inflammation contributes to an immunosuppressive microenvironment, promotes angiogenesis, impairs DNA repair, and activates androgen receptor signaling (8). Among inflammatory markers, the platelet-to-lymphocyte ratio (PLR) has gained attention for its low cost, reproducibility, and clinical accessibility. PLR reflects both thrombocytosis, which supports tumor survival and metastasis (9), and lymphopenia, which indicates weakened antitumor immunity (10), thus offering a dual perspective on tumor-host dynamics.

In CRPC, elevated PLR is strongly associated with aggressive tumor behavior and unfavorable prognosis. Neuberger M et al. (2023) (11) demonstrated that high PLR independently predicted shorter OS in individuals treated with docetaxel. Similarly, Wang ZP et al. (2024) (12) reported that elevated PLR during abiraterone therapy was associated with reduced OS and PFS. These findings highlight PLR as a low-cost, accessible prognostic marker in the management of CRPC.

Previous meta-analyses on PLR in prostate cancer often lacked stage-specific stratification. For instance, Guo JN et al. (2018) (13) included five studies and found that elevated PLR was associated with poorer OS and PFS but did not differentiate CRPC from other stages. Salciccia S et al. (2022) (14) analyzed metastatic and non-metastatic cases separately and found no association between PLR and biochemical progression or mortality in metastatic disease. These inconsistent results reflect the limited and inconclusive evidence for PLR in CRPC, highlighting the need for stage-specific analyses. CRPC’s distinct inflammatory microenvironment may uniquely affect the prognostic value of PLR.

To address these limitations, we conducted a systematic review and meta-analysis focusing on CRPC to evaluate the prognostic value of PLR for OS and PFS. Subgroup and mechanistic analyses were performed to explore sources of heterogeneity and underlying biological mechanisms. This analysis provides the most comprehensive evidence to date supporting PLR as a standardized risk stratification tool in CRPC, offering insights to guide the development of future biomarker-driven therapeutic strategies.

## 2 Materials and methods

### 2.1 Literature search

The present review adhered to the PRISMA 2020 guidelines for conducting and reporting systematic reviews (15), and the protocol was registered in the PROSPERO (CRD420251029427). Two investigators (CCZ and YLJ) independently developed the search strategy. They formulated search terms and keywords for use across multiple databases, including PubMed, Embase, Web of Science, and the Cochrane Library, covering publications up to March 11, 2025. The key search terms included “castration-resistant prostate cancer”, “platelet”, and “lymphocyte”. The comprehensive search strategy is provided in **Supplementary File 1**.

### 2.2 Study selection

Studies were included based on the following criteria: (1) patients had a confirmed diagnosis of CRPC; (2) all treatment modalities were considered eligible; (3) the study investigated the prognostic impact of PLR on OS or PFS; (4) HR with 95% CIs were either directly reported or derivable from available data; (5) patients were stratified into high and low PLR groups based on predefined cut-off values; (6) the study was fully published; and (7) the study design was either a randomized controlled trial or a cohort study.

Studies were excluded based on the following criteria: (1) commentaries, reviews, conference abstracts, case reports, or letters; (2) studies lacking sufficient data to calculate HRs and 95% CIs; (3) studies not reporting survival outcomes; and (4) duplicate or overlapping data.

Two reviewers (CCZ and YLJ) independently screened the titles and abstracts of all retrieved records, followed by full-text evaluation to confirm eligibility. All assessments were conducted independently and cross-checked. Discrepancies were resolved through discussion or, when necessary, by consulting a third reviewer (JHY).

### 2.3 Data extraction

Data extraction was independently performed by two reviewers (CCZ and YLJ). To ensure accuracy, all extracted data were cross-verified, with any discrepancies resolved through discussion or, if necessary, consultation with a third reviewer (JHY). The extracted variables included: the first author’s name, year of publication, country, study design, sample size, patient age, treatment regimen, PLR cut-off value, follow-up duration, and HRs (with 95% CIs) for OS and PFS.

### 2.4 Quality assessment

The quality of the studies was assessed using the NOS (16), which evaluates three domains: selection of study groups, comparability of cohorts, and outcome assessment, across a total of eight items. The maximum score is 9, with studies scoring 7 or higher considered to be of high quality.

### 2.5 Statistical analysis

Pooled HRs (with 95% CIs) were calculated to assess the prognostic significance of PLR in individuals with CRPC. When both univariate and multivariate analyses were provided in the same study, HRs from multivariate models were preferentially included, as they account for potential confounding exposures.

Heterogeneity among studies was assessed via Cochran’s Q test and Higgins’ I² statistic (17). Subgroup and sensitivity analyses were performed to evaluate the stability of results for OS and PFS and to explore potential sources of heterogeneity. Publication bias was evaluated using funnel plots and Egger’s test (18), with a p-value <0.05 denoting statistical significance. All analyses were conducted using STATA version 15.0 and Review Manager version 5.3.

## 3 Results

### 3.1 Study characteristics

**Figure 1:**
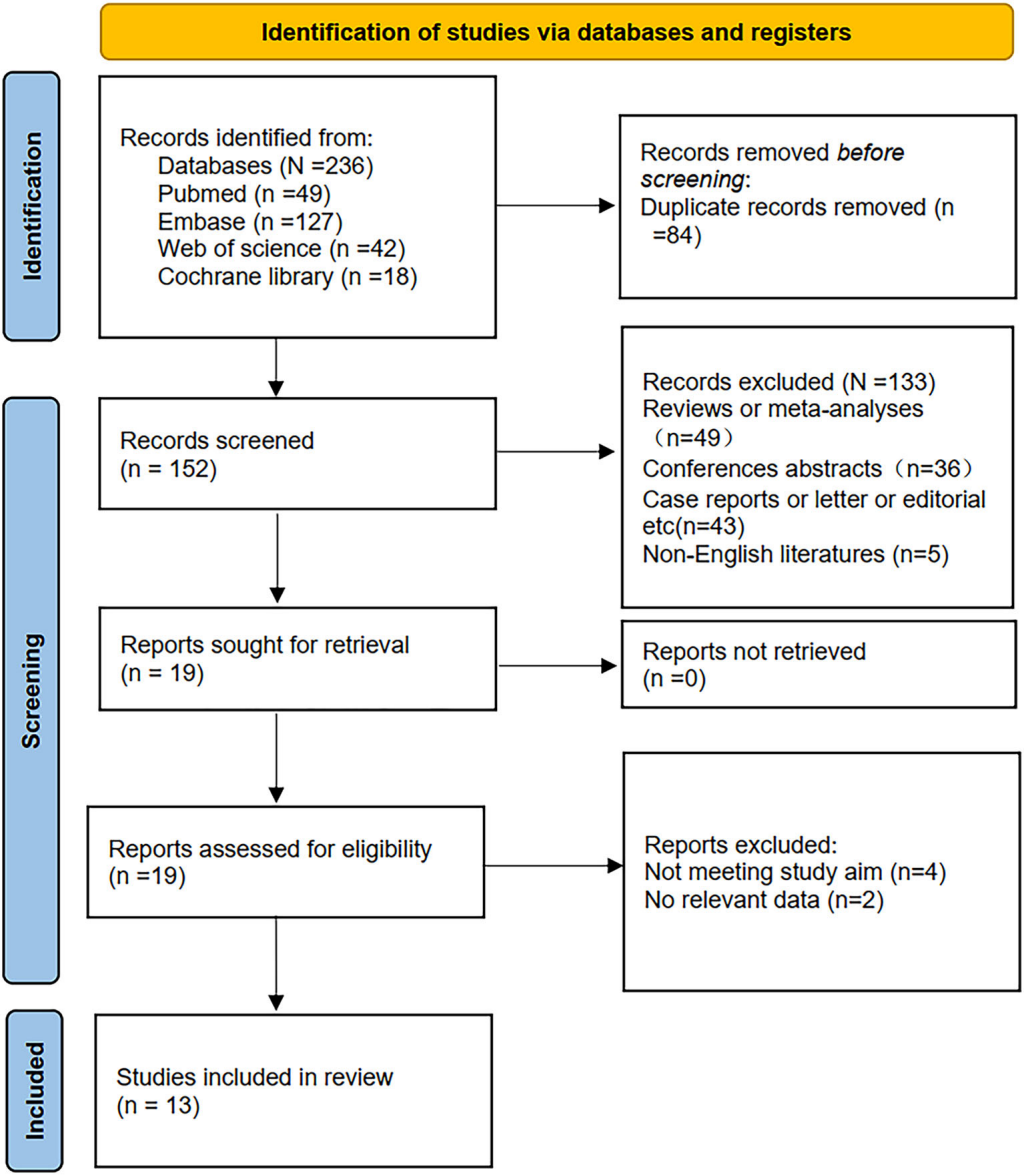
Flow of literature screening and inclusion.

Initially, 236 records were retrieved through database searches. Following the removal of 84 duplicates, 152 studies remained for title and abstract screening, leading to the exclusion of 133 irrelevant articles. The full texts of the remaining 19 publications were then assessed in detail, with 6 studies excluded due to insufficient survival data or inconsistent study objectives. Ultimately, 13 studies (11, 12, 19–29) comprising 2,405 patients were included in this meta-analysis (**Figure 1**).

Among the 13 eligible studies, one was conducted in Japan, one in Spain, one in the United States, one in Germany, two in Turkey, three in China, and four in Italy. Notably, one included article encompassed two independent cohort studies, resulting in 14 comparison groups. Of the included cohorts, nine employed a retrospective design, while four were conducted prospectively.

All studies, published in English between 2016 and 2024, evaluated patients stratified into high-PLR and low-PLR groups. Thirteen studies assessed the prognostic impact of PLR on OS, while four specifically investigated its association with PFS. All studies were graded with NOS scores ranging from 7 to 9, reflecting high methodological quality. A summary of the studies’ baseline characteristics and their respective NOS ratings is presented in **Table 1**.

**Table 1:** Baseline characteristics of the included patients.

Author	Year	Country	Type of study	N	Age	Stage	Treatment	PLR cut-off	Follow-up	NOS
Sahin E	2023	Turkey	retrospective	61	69.8	mCRPC	Lu-PSMA-617	134.27	53.2	7
Yamada Y (20)	2020	Japan	retrospective	196	75	mCRPC	abiraterone, enzalutamide or chemotherapy	66.88	40.12	7
Wang ZP (12)	2024	China	retrospective	158	71	mCRPC	abirateron	112.86	49.7	7
Shi XY (21)	2021	China	retrospective	399	68	mCRPC	docetaxel chemotherapy	104.28	36	8
Pisano C (22)	2021a	Italy	retrospective	225	73	mCRPC	abirateron or enzalutamide	128	NA	8
Pisano C (22)	2021b	Italy	retrospective	225	73	mCRPC	abirateron or enzalutamide	190	NA	8
Neuberger M (11)	2023	Germany	retrospective	118	72	mCRPC	docetaxel chemotherapy	233	18.5	7
Man YN (23)	2019	China	retrospective	179	70	mCRPC	docetaxel chemotherapy	210	24	7
Lolli C (24)	2016	Italy	retrospective	230	74	mCRPC	abirateron	210	29	7
Donate-Moreno MJ (25)	2022	Spain	prospective	80	70.2	mCRPC	abirateron or enzalutamide	150	19	7
Chong W (26)	2021	USA	retrospective	63	70.9	mCRPC	abiraterone, enzalutamide or chemotherapy	155.54	17.6	7
Onal C (27)	2019	Turkey	prospective	102	71	mCRPC	abirateron	163	24	9
Bauckneht M (28)	2022	Italy	prospective	519	74	mCRPC	radium-223	145.9	10.7	7
Angusti T (29)	2022	Italy	prospective	75	73	mCRPC	radium-223	NA	45.5	7

### 3.2 Meta-analysis results

#### 3.2.1 PLR and overall survival

**Figure 2:**
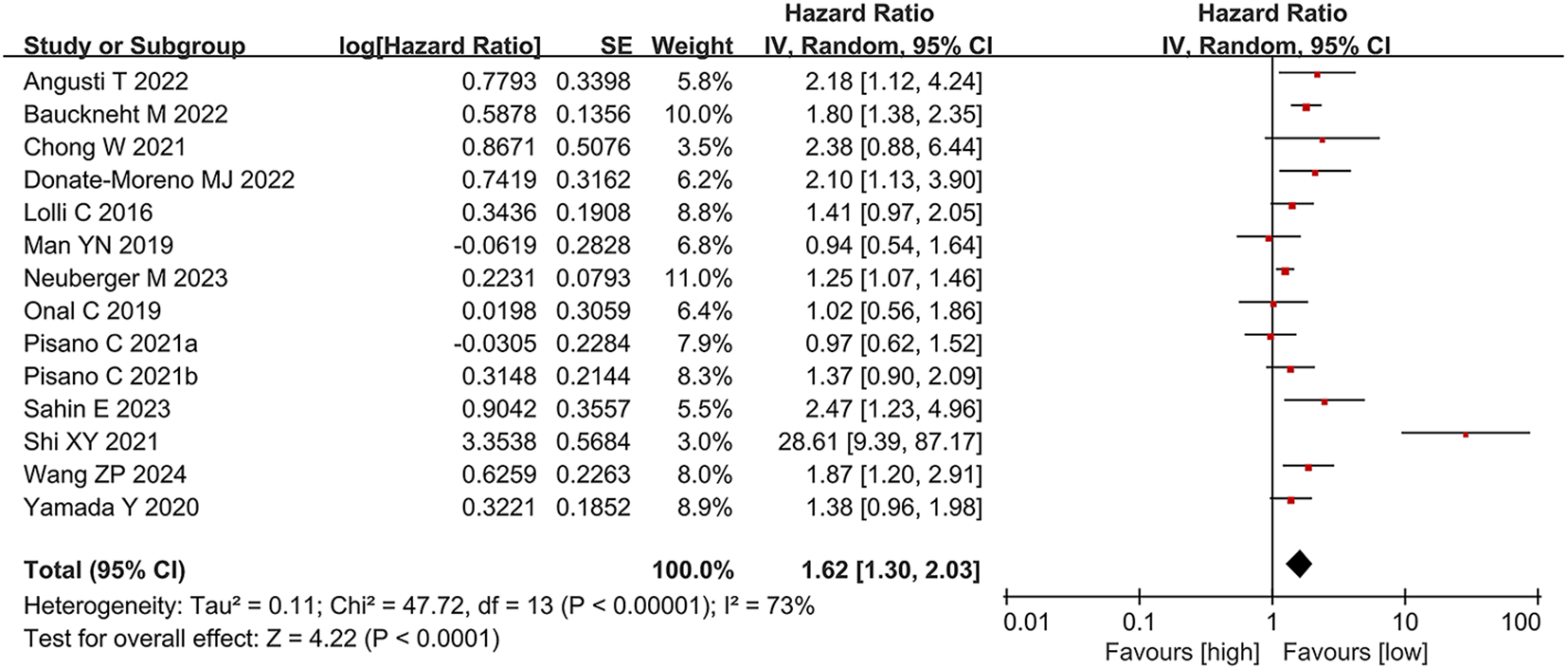
Forest plot for OS.

We analyzed the association between PLR and OS across 13 studies (14 comparison groups) involving 2,405 participants. Substantial heterogeneity was detected among the publications (I² = 73%, p < 0.00001). The pooled analysis demonstrated that elevated PLR was significantly linked to shorter OS in individuals with CRPC (HR = 1.62, 95% CI: 1.30-2.03; p < 0.0001) (**Figure 2**).

Subgroup analyses were conducted based on geographic region, treatment regimen, study design, age, and PLR cut-off values to explore potential sources of heterogeneity. The results of these analyses are summarized in **Table 2**. Heterogeneity appeared to be influenced by region, age, study design, treatment modality, and cut-off values. Notably, no significant link was observed between PLR and OS in the American subgroup and the chemotherapy subgroup; however, the association remained significant in all other subgroups.

**Table 2:** Subgroup analysis.

Subgroup	OS			
	Study group	HR [95%CI]	*P* value	*I* ^2^
Total	14	1.62 [1.30-2.03]	<0.0001	73%
Study design				
Prospective	4	1.72[1.31-2.27]	<0.0001	23%
Retrospective	10	1.63 [1.22-2.17]	0.001	77%
Mean/median age				
≥72y	7	1.40 [1.19-1.64]	<0.0001	37%
<72y	7	2.26 [1.25-4.08]	0.007	82%
Region				
Asia	4	2.44 [1.05-5.71]	0.04	90%
Europe	9	1.46 [1.21-1.75]	<0.0001	47%
America	1	2.38 [0.88-6.44]	0.09	NA
PLR cut-off				
≥150	7	1.29[1.13-1.47]	0.0001	2%
<150	6	2.13 [1.31-3.47]	0.002	85%
Treatment				
Hormonal therapy	6	1.39 [1.11-1.74]	0.004	28%
Chemotherapy	3	2.75 [0.82-9.15]	0.1	94%
Radiopharmaceutical	3	1.91 [1.51-2.41]	<0.0001	0%
Combination therapy	2	1.48 [1.04-2.10]	0.03	2%

#### 3.2.2 PLR and progression-free survival

**Figure 3:**
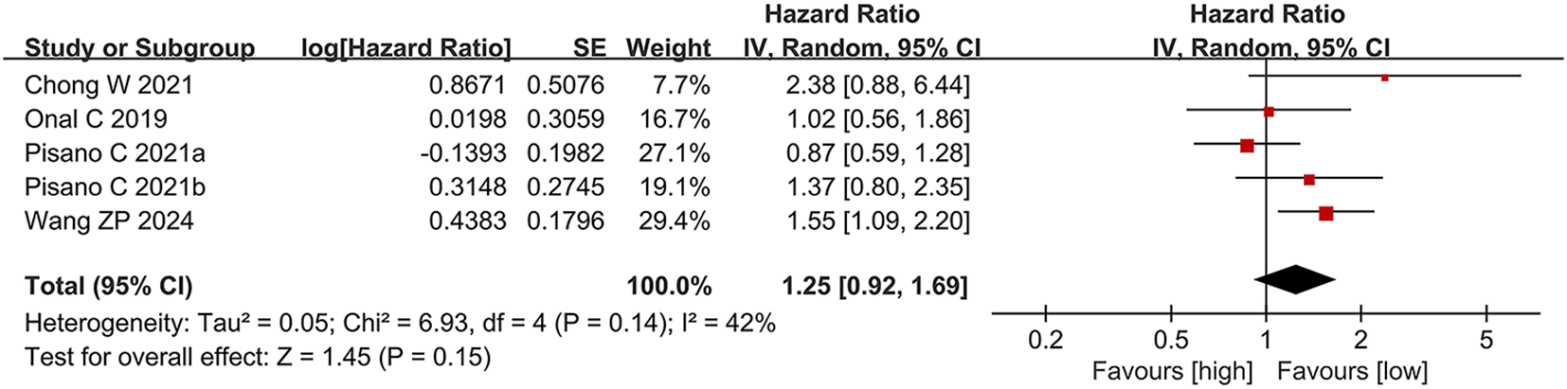
Forest plot for PFS.

Four studies (12, 22, 26, 27), comprising five comparison groups, reported data on the link between PLR and PFS. In contrast to the findings for OS, elevated PLR was not significantly linked to PFS in individuals with CRPC (HR = 1.25, 95% CI: 0.92-1.69; p = 0.15). No significant heterogeneity was detected among these publications (I² = 42%, p = 0.14) (**Figure 3**).

### 3.3 Sensitivity analysis

Sensitivity analyses were performed to evaluate the robustness of the results related to baseline PLR and its clinical relevance. Sequential exclusion of each individual study revealed consistent effect estimates within the original confidence interval range, indicating that no individual study disproportionately affected the pooled results for OS or PFS (**Supplementary File 2**). These findings confirm the stability and reliability of the meta-analysis results.

### 3.4 Publication bias

Publication bias was assessed through funnel plots and Egger’s test. For the meta-analysis of OS, neither the funnel plot nor Egger’s test demonstrated significant publication bias (Egger’s p = 0.067). Similarly, no significant publication bias was detected in the PFS meta-analysis (Egger’s p = 0.53) (**Supplementary File 2**).

## 4 Discussion

In recent years, the association between systemic inflammatory responses and cancer prognosis has garnered increasing attention. The PLR, an integrative inflammatory marker, has emerged as a promising prognostic indicator due to its simplicity, low cost, and widespread availability. PLR has been shown to correlate strongly with patient survival in various solid tumors, including colorectal cancer (30), lung cancer (31), and endometrial cancer (32). However, in CRPC, the terminal stage of prostate cancer, the prognostic value of PLR remains controversial.

Findings from the meta-analysis of 13 studies involving 2,405 patients indicated that increased PLR was significantly linked to shorter OS in CRPC (HR = 1.62), while no significant association was observed with PFS. A prior meta-analysis by Guan YP et al. (2020) (33), based on only three studies, reported worse OS in metastatic CRPC patients with elevated PLR receiving abiraterone or enzalutamide. Compared to earlier studies that focused mainly on hormone-treated patients, our study includes more studies and a broader range of treatments, providing a clearer view of PLR’s stratification value in advanced CRPC. PLR appears to be a more consistent predictor of long-term outcomes like OS. At the same time, its association with short-term endpoints such as PFS may be influenced by treatment variability and tumor heterogeneity. This interpretation should be viewed with caution because only four studies reported PFS and the corresponding effect estimate was likely underpowered. By contrast, for overall survival we were able to include a larger number of CRPC studies and to perform rigorous subgroup and sensitivity analyses, so our study strengthens the evidence supporting the prognostic value of PLR in CRPC, particularly for OS.

Subgroup analyses provided new insights into the heterogeneous prognostic value of PLR in prostate cancer. Regionally, no significant association was found between PLR and OS in the Americas (HR = 2.38, p = 0.09), likely due to the limited statistical power resulting from a single study (n = 158) and biological variability associated with ethnic diversity. In contrast, higher PLR was consistently linked to poorer OS in both Asian (HR = 1.71, I² = 90%) and European (HR = 1.54, I² = 47%) populations, supporting its cross-ethnic applicability. The high heterogeneity, particularly in Asian cohorts, may reflect underlying biological complexity, including genetic variations that influence inflammatory thresholds, platelet activation, and lymphocyte function (34, 35). Healthcare disparities may also contribute. In developed countries like the U.S., widespread prostate cancer screening often identifies early-stage cases, while studies from developing regions include more advanced disease, potentially confounding the PLR–prognosis relationship (36, 37). In the treatment-based analysis, PLR showed no significant association with OS in the chemotherapy subgroup (HR = 2.75, p = 0.10). This may be due to the bone marrow-suppressive effects of docetaxel (38), which impairs both platelet and lymphocyte production by disrupting microtubule function and inducing hematopoietic stem cell arrest. These effects can lead to asynchronous cell count fluctuations, reducing PLR stability and interpretability (39). Similar hematologic toxicity profiles were reported in pivotal docetaxel trials in mCRPC, where neutropenia and other cytopenias were frequent (40, 41), supporting the notion that chemotherapy itself can distort inflammation-based ratios. Therefore, the null finding in the chemotherapy subgroup may reflect treatment-related hematologic interference rather than a true absence of prognostic value. Future studies should incorporate serial PLR measurements (baseline, on-treatment, and post-treatment) to better define its prognostic dynamics in docetaxel-treated CRPC.

Inflammatory responses in the tumor microenvironment are closely linked to cancer progression and metastasis (42). Platelets, as key effector cells, may drive CRPC progression through several mechanisms. They release VEGF and PDGF, promoting angiogenesis via endothelial receptor activation and supporting tumor growth and metastasis (43), with pro-angiogenic activity being strongly associated with bone metastases (44). Platelet-derived TGF-β1 also induces epithelial–mesenchymal transition (EMT) through the Smad2/3 pathway, enhancing tumor cell migration and invasion (45). In addition, circulating tumor cells (CTCs) can bind to platelets, forming protective complexes that shield them from NK cell–mediated cytotoxicity and facilitate vascular adhesion (46). In the tumor immune microenvironment, elevated PLR is often associated with reduced CD8^+^ T-cell counts (47), which are linked to poor prognosis in metastatic CRPC (10). Tumor-associated macrophages (TAMs) suppress CD8^+^ T-cell proliferation by secreting IL-10 and PGE2 (48, 49). Myeloid-derived suppressor cells (MDSCs) further impair antitumor immunity by depleting L-arginine via arginase-1 (Arg-1), leading to T-cell dysfunction (50).

The chronic inflammatory microenvironment drives tumor metastasis through several signaling pathways. IL-6/STAT3 activation enhances resistance to apoptosis and promotes cancer cell proliferation, invasion, and metastasis (51). The TNF-α/NF-κB pathway accelerates the tumor cell cycle and growth (52). Additionally, the COX-2/PGE2 axis suppresses immune responses and supports tumor stemness through receptor-mediated signaling (53). These immune–inflammatory pathways provide a plausible biological explanation for the association between elevated PLR and poorer outcomes in CRPC. In addition, about 20%–30% of metastatic or castration-resistant cases carry somatic or germline DNA damage response (DDR) alterations, with BRCA2 among the most frequent, and these changes are also associated with more aggressive disease (54–56). Because most included studies did not report such genomic data, we could not determine how much of the risk attributed to PLR overlaps with this unmeasured high-risk molecular subset, which should be clarified in genomically profiled cohorts.

This study has several limitations. First, although 13 studies were included, only three studies included patients treated with chemotherapy, and only four studies reported PFS, which reduces the statistical power of these subgroup analyses and may partly account for the non-significant findings. Second, most of the included studies were retrospective and used different PLR cut-off values and outcome definitions, so residual confounding and methodological heterogeneity cannot be excluded; moreover, inflammatory comorbidities such as chronic infections or autoimmune diseases were not reported in the original studies, so we were unable to assess their potential impact on PLR independent of tumor burden. Third, genomic information relevant to advanced prostate cancer was not reported in most primary studies, so we could not adjust for potentially important molecular prognostic factors. Finally, most cohorts originated from Europe and Asia, with limited representation from other regions, which may restrict the generalizability of our conclusions. Prospective, multicenter, genomically annotated studies with standardized and, ideally, longitudinal PLR assessment are needed to validate these results.

In summary, this meta-analysis confirms that elevated PLR is an independent adverse prognostic factor for OS in CRPC, with consistent findings in hormone therapy subgroups and European populations. Despite variability in cut-off values, PLR’s low cost and accessibility support its potential role in clinical risk stratification. Future studies should standardize measurement protocols and explore the combination of PLR with other markers, such as the neutrophil-to-lymphocyte ratio (NLR), to develop multiparametric prognostic models. Further investigation into PLR’s association with immunotherapy response is also warranted. These efforts could help shift PLR from a prognostic marker to a tool for guiding clinical decisions, advancing precision management in CRPC.

## 5 Conclusion

This meta-analysis confirms that PLR is a significant prognostic marker for OS in CRPC, with higher levels associated with shorter OS. However, its prognostic value was not significant among patients receiving chemotherapy or in American populations. Given the heterogeneity and limitations of retrospective studies, prospective multicenter research is necessary to validate these findings. Standardizing PLR measurement and investigating its interaction with emerging therapies such as PARP inhibitors and immunotherapy will be essential for refining its clinical applicability. Ultimately, incorporating PLR into prognostic models may facilitate early identification of high-risk patients and guide timely, targeted treatment.

## Data Availability

The raw data supporting the conclusions of this article will be made available by the authors, without undue reservation.
