# Supplementary material for: Prognostic value of platelet to lymphocyte ratio in patients with castration-resistant prostate cancer: a systematic review and meta-analysis
Source: Front Oncol. 2025 Dec 11;15:1655520. doi: 10.3389/fonc.2025.1655520 (PMC12739549; doi:10.3389/fonc.2025.1655520)
Supplement: Supplementary file 1 [file DataSheet1.docx]

**Supplementary Materials**

**Supplementary file 1:** The search strategy.

| **Databases** | **Searching strategy** | **Total** |
| --- | --- | --- |
| PubMed | (((Blood Platelets[MeSH Terms]) OR ((((Platelets) OR (Platelet)) OR (Thrombocytes)) OR (Thrombocyte))) AND ((Lymphocytes[MeSH Terms]) OR (((((Lymphocyte) OR (Lymphoid Cells)) OR (Lymphoid Cell)) OR (immune competent cell)) OR (lymph cell)))) AND ((Castration-Resistant Prostatic Neoplasms[MeSH Terms]) OR (((((((((((((((((((((((((((((((androgen independent PC) OR (androgen independent PCa)) OR (androgen independent prostate cancer)) OR (androgen independent prostatic cancer)) OR (Androgen Independent Prostatic Cancers)) OR (Androgen Independent Prostatic Neoplasms)) OR (androgen insensitive PC)) OR (androgen insensitive PCa)) OR (androgen insensitive prostate cancer)) OR (androgen insensitive prostatic cancer)) OR (Androgen Resistant Prostatic Cancer)) OR (castrate refractory prostate cancer)) OR (castrate resistant PC)) OR (castrate resistant PCa)) OR (castrate resistant prostate cancer)) OR (castration refractory PCa)) OR (castration refractory prostate cancer)) OR (castration resistant PC)) OR (castration resistant PCa)) OR (castration resistant prostate cancer)) OR (castration resistant prostatic cancer)) OR (Castration Resistant Prostatic Neoplasm)) OR (castration resistant prostatic neoplasms)) OR (CRPC)) OR (hormone refractory PC)) OR (hormone refractory PCa)) OR (hormone refractory prostate cancer)) OR (hormone refractory prostatic cancer)) OR (hormone resistant PCa)) OR (hormone resistant prostate cancer)) OR (hormone resistant prostatic cancer))) | 49 |
| Web of science | (((Blood Platelets) OR ((((Platelets) OR (Platelet)) OR (Thrombocytes)) OR (Thrombocyte))) AND ((Lymphocytes) OR (((((Lymphocyte) OR (Lymphoid Cells)) OR (Lymphoid Cell)) OR (immune competent cell)) OR (lymph cell)))) AND ((Castration-Resistant Prostatic Neoplasms) OR (((((((((((((((((((((((((((((((androgen independent PC) OR (androgen independent PCa)) OR (androgen independent prostate cancer)) OR (androgen independent prostatic cancer)) OR (Androgen Independent Prostatic Cancers)) OR (Androgen Independent Prostatic Neoplasms)) OR (androgen insensitive PC)) OR (androgen insensitive PCa)) OR (androgen insensitive prostate cancer)) OR (androgen insensitive prostatic cancer)) OR (Androgen Resistant Prostatic Cancer)) OR (castrate refractory prostate cancer)) OR (castrate resistant PC)) OR (castrate resistant PCa)) OR (castrate resistant prostate cancer)) OR (castration refractory PCa)) OR (castration refractory prostate cancer)) OR (castration resistant PC)) OR (castration resistant PCa)) OR (castration resistant prostate cancer)) OR (castration resistant prostatic cancer)) OR (Castration Resistant Prostatic Neoplasm)) OR (castration resistant prostatic neoplasms)) OR (CRPC)) OR (hormone refractory PC)) OR (hormone refractory PCa)) OR (hormone refractory prostate cancer)) OR (hormone refractory prostatic cancer)) OR (hormone resistant PCa)) OR (hormone resistant prostate cancer)) OR (hormone resistant prostatic cancer))) (Topic) | 42 |
| Embase | ((Blood Platelets or (Platelets or Platelet or Thrombocytes or Thrombocyte)) and (Lymphocytes or (Lymphocyte or Lymphoid Cells or Lymphoid Cell or immune competent cell or lymph cell)) and (Castration-Resistant Prostatic Neoplasms or (androgen independent PC or androgen independent PCa or androgen independent prostate cancer or androgen independent prostatic cancer or Androgen Independent Prostatic Cancers or Androgen Independent Prostatic Neoplasms or androgen insensitive PC or androgen insensitive PCa or androgen insensitive prostate cancer or androgen insensitive prostatic cancer or Androgen Resistant Prostatic Cancer or castrate refractory prostate cancer or castrate resistant PC or castrate resistant PCa or castrate resistant prostate cancer or castration refractory PCa or castration refractory prostate cancer or castration resistant PC or castration resistant PCa or castration resistant prostate cancer or castration resistant prostatic cancer or Castration Resistant Prostatic Neoplasm or castration resistant prostatic neoplasms or CRPC or hormone refractory PC or hormone refractory PCa or hormone refractory prostate cancer or hormone refractory prostatic cancer or hormone resistant PCa or hormone resistant prostate cancer or hormone resistant prostatic cancer))) | 127 |
| Cochrane | ((Blood Platelets or (Platelets or Platelet or Thrombocytes or Thrombocyte)) and (Lymphocytes or (Lymphocyte or Lymphoid Cells or Lymphoid Cell or immune competent cell or lymph cell)) and (Castration-Resistant Prostatic Neoplasms or (androgen independent PC or androgen independent PCa or androgen independent prostate cancer or androgen independent prostatic cancer or Androgen Independent Prostatic Cancers or Androgen Independent Prostatic Neoplasms or androgen insensitive PC or androgen insensitive PCa or androgen insensitive prostate cancer or androgen insensitive prostatic cancer or Androgen Resistant Prostatic Cancer or castrate refractory prostate cancer or castrate resistant PC or castrate resistant PCa or castrate resistant prostate cancer or castration refractory PCa or castration refractory prostate cancer or castration resistant PC or castration resistant PCa or castration resistant prostate cancer or castration resistant prostatic cancer or Castration Resistant Prostatic Neoplasm or castration resistant prostatic neoplasms or CRPC or hormone refractory PC or hormone refractory PCa or hormone refractory prostate cancer or hormone refractory prostatic cancer or hormone resistant PCa or hormone resistant prostate cancer or hormone resistant prostatic cancer))) | 18 |

**Supplementary file 2:** Sensitivity analysis and publication bias.


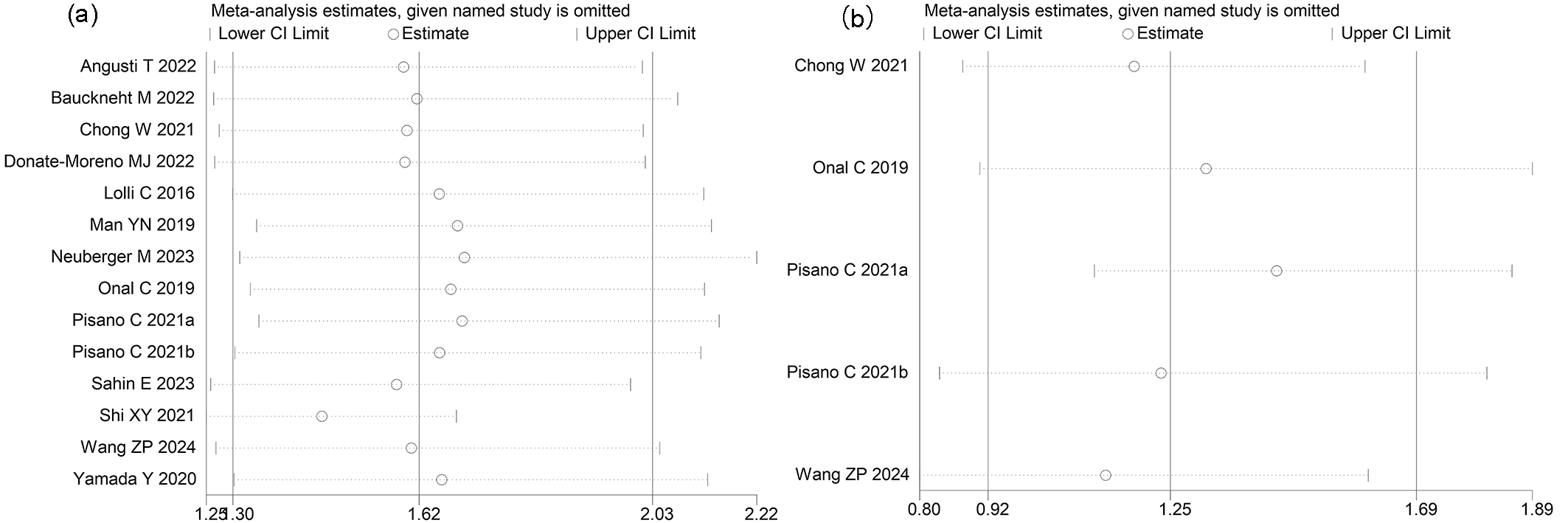


**Figure. S1** Sensitivity analysis of (a) OS(a) and (b) PFS.


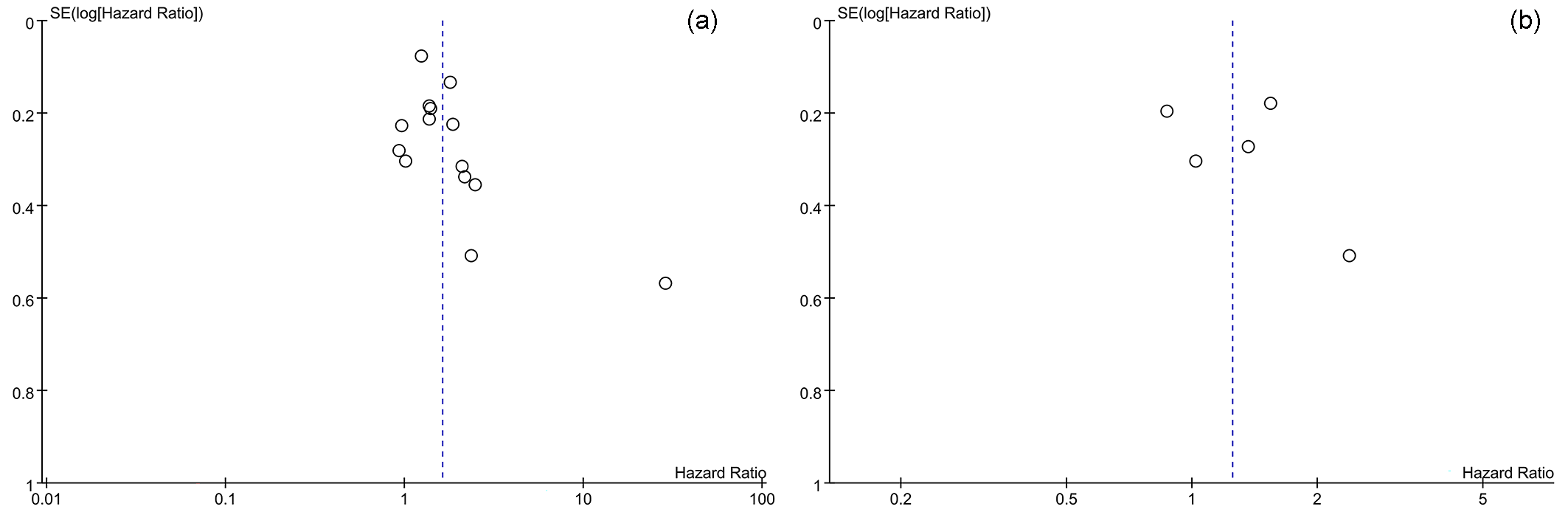


**Figure. S2** Funnel plot for publication bias of (a) OS and (b) PFS.
